# Intergenerational Socioeconomic Mobility and Cognitive Impairment in Chinese Older Adults: Does Gender Matter?

**DOI:** 10.1093/geroni/igab046.2627

**Published:** 2021-12-17

**Authors:** Rong Fu, Yujun Liu

**Affiliations:** 1 Siena College, Loudonville, New York, United States; 2 Northern Illinois University, Naperville, Illinois, United States

## Abstract

The prevalence of dementia among older adults in mainland China is projected to increase rapidly in the next few decades. This study aimed to examine the impact of intergenerational socioeconomic mobility on the risk of cognitive impairment in a cohort of Chinese older adults, with a focus on potential gender differences. Data were derived from the 2011 wave of the Chinese Longitudinal Healthy Longevity Survey. Socioeconomic mobility in this study includes three dimensions: occupational mobility, educational mobility, and residential mobility. Cognitive impairment was assessed using the Chinese version of Mini-Mental State Examination. The final sample included 6,233 older adults aged 80 years and above. Logistic regression models were performed to assess the impact of the three dimensions of socioeconomic mobility on the risk of cognitive impairment in older men and women. For men, those with stable high occupational status across generations had the lowest risk of cognitive impairment. For women, those who received no education and lived in rural areas across generations had the highest risk of cognitive impairment. These findings lend support to the cumulative risk theory, which highlights the accumulation of risk factors that places individuals in jeopardy for negative health consequences in later life. The findings have implications for advancing supportive policies and practices related to maximizing the benefits of education and occupation for cognition in later life, especially for women in rural China.

